# The Teeth of the Horse and Their Diseases

**Published:** 1882-04

**Authors:** R. Jennings


(*To be continued.*)

---

### ART. XII.—THE TEETH OF THE HORSE, AND THEIR DISEASES.

BY R. JENNINGS, V.S.

I have chosen this subject, believing it may possess sufficient interest to render it acceptable to many readers who may be interested in equine stock, and for the purpose of putting upon record a few facts, the result of my own experience and investigation, and which appear to have been overlooked or ignored by veterinary writers. My will is good, but I feel myself incompetent to do this subject the justice which I believe it fully merits. I shall, however, attempt to show a few practical points which, to me, seem of importance to the veterinary student, believing that my theory will bear the closest scrutiny, and receive the endorsement of honest investigation. Dentition belonging to the class mammalia is the most important division of the digestive apparatus, which, as a whole, is the most complete of the several organs into which the animal frame is divided, and upon which we are dependent for existence. The digestive organs in the biped and the quadruped are varied, in order to adapt each to its natural instincts, and the teeth to assume different forms in different species of animals. The order and precision with which this has been effected enables the comparative anatomist, from the inspection of a single tooth, to describe the habits and peculiarities of the animal to which it belonged, whether carnivorous or herbivorous. This admirable arrangement of the teeth enables the animal to gather its food and convert substances quite different in their structure into material which nourish and build up the animal frame. The consideration of the embryotic formation of the teeth I do not consider of sufficient importance, in connection with this subject, for encroaching upon your valuable space; will therefore pass on to the consideration of the several divisions of the teeth, and the order in which they make their appearance in the mouth during the process of dentition. In the mature horse, we find forty teeth, namely, twelve incisors, or nippers, situated in the anterior portion of the mouth, six in the upper and six in the lower jaw. Their use is to seize their food, cut and take it into the mouth, where it undergoes the process of trituration by the grinding of the molar teeth, which are twenty-four in number; twelve in each jaw, and situated on either side of the face. The cuspidati, canine teeth or tushes, are four in number, and situated two in each jaw, between the incisor and molar teeth. The colt, during the natural process of dentition, cuts and sheds off twenty-six teeth, viz.: Twelve incisor teeth, arranged as in the adult horse, the receptacle for which is the alveolus of the anterior superior maxillary bones, which are divided by nature at the median line. Twelve molar teeth are alike situated in the posterior superior maxillary bones, which give shape and solidity to the cheeks. This arrangement differs from the anatomical divisions of the bones of the face in the human being, inasmuch as there are four superior maxillary bones in the equine animal, while in man there are but two. These bones in both species of animals largely make up the anterior and lateral portions of the face. The foal, at birth, usually has all the temporary molar teeth, and four of the incisors, which are the central pairs, one pair in the upper and one in the lower jaw. During the second month, the second pair of incisor teeth make their appearance, one in the upper and one in the lower jaw, on either side of the first or central pairs. About the eighth month of the animal's life, the third and last pairs make their appearance, one above and one below, on either side of the second pairs; these complete the set of incisors. The wolf teeth make their appearance between the first and second year, and are situated immediately anterior to the first molar teeth of the upper jaw, on either side. At the age of two years, the deciduous teeth are fully developed, the colt having a full mouth. Mayhew says: "The wolf's teeth at the anterior of the two rows of upper molars are generally present or indicated at birth; but, as they are not invariably found, no distinct notice need be taken of their existence." This opinion evidently is not based upon personal experience or investigation, but is the result of casual observation only. From the year 1859 to 1867, I had the medical charge of the brood farm of Adolph Malliard, Esq., Bordentown, New Jersey. Upon this farm an average of more than two hundred blooded animals were constantly kept, which afforded me many opportunities for investigation, all of which I improved. In every instance, where the foal was lost in parturition, natural or premature (by premature I do not mean abortion), I examined the jaw-bones of the foal, and in every instance found the alveolus of the wolf teeth distinct and filled with the pulp of the future teeth. But in no case did I find these teeth developed. Various are the theories which have from time to time been advanced regarding these teeth, arising, for the most part, from want of proper investigation and the assertions of writers founded upon theories born in their versatile imagination, which the following will fully prove: "Every Man his own Farrier," page 201: "This is a small tooth appearing on the upper jaw, at a distance of about half an inch from the grinders, sometimes on one side and at other times on both sides. These teeth are seldom found in young horses, but old horses are sometimes subject to them. They are supposed to affect the eyes at different times; they must be removed either by application of a hammer and chisel made for the purpose, or by filing them down level with the gums." The author of the above places these teeth in a new position. This reminds me of one J. Z. Walling, a veterinary dentist, who, in a communication published in an agricultural paper, says: "The horse cuts the canine teeth at the age of five years, and sheds them about the eleventh year." Comment is unnecessary. It is quite evident that in the days of Gibson, Bracken, Bartlett and other writers of their day, that these teeth had not been noticed, as the following quotation will fully prove: "Wolves' Teeth.—A horse is said to have wolves' teeth when the teeth grow in such a manner that their points prick or wound either the tongue or gums in eating. Old horses are most liable to this infirmity, and whose upper teeth overshoot the under ones in a great degree. To remedy this evil you may either chop off the superfluous parts of the teeth with a chisel and mallet, or file them down, which is the better way" The condition of the teeth here referred to will come under our notice as we proceed. But to return to our subject. These teeth are natural to all colts, and are developed in the same manner as the other teeth; yet few veterinary authors regard them of sufficient importance to mention them in their works. The germs of these teeth are found in the foal at birth, developed in the jaws of the yearling, ready to make their way through the gums, and do appear in the mouth of all colts at some period between the first and the third year of its age. They will be found in a large majority of colts at the age of two years. In the examination of more than one hundred colts' jaws, during an active practice of thirty-seven years, which have died under two years of age, not a single instance occurred where these teeth, or the germs which produce them, were not found; proving, beyond a doubt, their existence as natural teeth belonging to the class deciduous.

